# Acute neuronal cell death and neuroinflammation per se do not trigger secondary autoimmune encephalitis in mice

**DOI:** 10.1038/s41598-025-08035-w

**Published:** 2025-06-27

**Authors:** Justus Wilke, Antonios Ntolkeras, Vinicius Daguano Gastaldi, Kathrin Borowski, Bianca Teegen, Winfried Stöcker, Fred Lühder, Klaus-Armin Nave, Hannelore Ehrenreich

**Affiliations:** 1 Clinical Neuroscience, Max Planck Institute for Multidisciplinary Sciences, City Campus, Göttingen, Germany; 2 Georg-August-University, Göttingen, Germany; 3 Clinical Immunological Laboratory Prof. H.C. (RCH) Winfried Stöcker, Groß Grönau, Germany; 4 Institute of Neuroimmunology and Multiple Sclerosis Research, University Medical Center of the Georg August University, Göttingen, Germany; 5 Department of Neurogenetics, Max Planck Institute for Multidisciplinary Sciences, City Campus, Göttingen, Germany; 6 Experimental Medicine, Department of Psychiatry and Psychotherapy, Central Institute of Mental Health, Medical Faculty Mannheim, Heidelberg University, J 5, 68159 Mannheim, Germany; 7 Present Address: Vollum Institute, Oregon Health and Science University, Portland, OR USA

**Keywords:** Acute encephalitis, Autoimmune relapse, Microglia, Autoimmunity, Autoantibodies, Autoimmunity, Molecular medicine, Neuroimmunology, Encephalopathy

## Abstract

Patients with virus encephalitis, such as herpes simplex encephalitis and Japanese encephalitis frequently relapse with autoimmune encephalitides associated with neural autoantibodies. It has been hypothesized that the infection-induced damage to the central nervous system results in shedding of neural autoantigens, their presentation to the peripheral immune system, and initiation of a secondary autoimmune encephalitis that targets these autoantigens. To test this hypothesis, we utilized a transgenic mouse model of virus-like but sterile encephalitis. After induction of acute neuronal death in the hippocampus, we monitored the mice for encephalitis-like symptoms for up to 10 months, evaluated the degree of neuroinflammation at several time points and screened their plasma for autoantibodies against 49 different autoimmune disease-associated brain autoantibodies. Throughout the study period, we did not detect any symptoms of severe autoimmune encephalitis, like hyperactivity, circling, seizures, lethargy. Evaluation of microglia numbers and morphology revealed pronounced microgliosis 1-week after initial encephalitis induction, which decreased over time. Scattered lymphocyte infiltration was present at all times in hippocampi of encephalitis mice, and did not increase over time. Perivascular cuffs were not detected. Infiltrating lymphocytes mainly consisted of CD8+ T cells. B cell infiltration was rare and did not differ from healthy control mice. High-parameter immunophenotyping of peripheral blood leukocytes did not reveal any changes associated with an autoimmune response. Testing all plasma samples (n = 30/group) at a dilution of 1:100 for autoantibodies against 49 neural autoantigens gave only two positive results, namely one healthy control with anti-CASPR2 autoantibodies (IgG) and one post-encephalitis mouse with anti-homer-3 autoantibodies (IgM). Overall, these findings suggest that acute neuronal cell death and neuroinflammation per se are not sufficient to trigger downstream autoimmune encephalitis relapses in mice.

## Introduction

Autoimmune-mediated clinical symptoms and autoantibodies against neuronal surface antigens are frequently observed in about one quarter of patients recovering from herpes simplex encephalitis (HSE) and Japanese encephalitis (JE)^1–15^. This secondary encephalitis is often referred to as autoimmune relapse or autoimmune encephalitis and typically occurs within 1–2 months after the primary virus encephalitis. It is characterized by an absence of the original pathogen, the presence of anti-neuronal autoantibodies, and autoimmune encephalitis-like symptoms that frequently improve after immunosuppressive treatment^7,10,12,13^. While most cases appear to be associated with autoantibodies against NMDA receptors, other anti-neuronal antibodies, including GABA_A_R, AMPAR, D2R-AB, are also detected in CSF of symptomatic and convalescent patients after HSE and JE^2,3,5–7,9,13,15,16^. It has been hypothesized that the virus-induced neuronal cell death, which is common in HSE and JE^14,17,18^, results in a release of neuronal surface antigens. These antigens are supposedly drained to peripheral lymphoid organs, where they are presented to the adaptive immune system, eliciting an autoimmune response against neuronal autoantigens. Presumably, activated autoreactive B cells, T cells, and antibody secreting cells then traffic to the CNS and induce a second neuroinflammatory response^19–23^. This hypothesis is supported by the absence of anti-neuronal autoantibodies during the acute phase of JE and HSE^7,13^, which indicates de novo autoantibody formation in response to encephalitis.

The central nervous system (CNS) has long been considered an immune-privileged site, but accumulating evidence suggests that its cellular components can differentially shape immune responses. A landmark study by Traka et al.^24^ demonstrated that targeted ablation of oligodendrocytes using a diphtheria toxin (DTA)-based approach in transgenic mice leads to delayed but sustained secondary autoimmune inflammation resembling multiple sclerosis. These findings suggest that oligodendrocyte death may not only be a consequence of CNS inflammation but also serve as a trigger for autoimmunity under certain conditions.

To date, it has remained unclear whether the selective loss of other CNS cell types can initiate a similar cascade. In particular, the immunological consequences of targeted neuronal ablation have not been systematically explored. Given the widespread neuronal loss observed in HSE and JE, as well as various neurodegenerative and autoimmune diseases, this question bears significant clinical relevance.

Here, we investigated whether selective ablation of neurons in adult mice using an analogous DTA-mediated system leads to the induction of CNS-targeted autoimmunity. In stark contrast to the oligodendrocyte ablation model, we found that neuroinflammation gradually subsided after targeted neuronal loss without detectable peripheral immune activation or relapse of CNS inflammation. Our findings suggest that the immunogenic potential of cell death within the CNS is not uniform across cell types and point to a unique role for oligodendrocytes in modulating neuroimmune interactions. Furthermore, the results suggest that additional factors, such as the prominent activation of the innate and adaptive immune system by virus/pathogen-associated molecular pattern pathways, are required to drive post-encephalitis autoimmune relapses.

## Methods

### Mice

All animal experiments were approved by the local animal care and use committee (LAVES, Niedersaechsisches Landesamt für Verbraucherschutz und Lebensmittelsicherheit, Oldenburg, Germany; license number 33.19-42502-04-18/2803) in accordance with the German animal protection law. The experiments were performed in accordance to approved protocols and reported following the ARRIVE guidelines. Mice were housed in groups of up to 16 in a temperature (~ 22 °C) and humidity (~ 50%) controlled environment, 12h light/dark cycle with food (standard food, Sniff Spezialdiäten, Germany) and water ad libitum. Cages were equipped with wood-chip bedding and nesting material (Sizzle Nest, Datesand, United Kingdom). Experimental mice were bred in-house (Max-Planck-Institute for Multidisciplinary Sciences) and weaned and separated by sex at postnatal day 21. Mice were randomly allocated to treatment groups (tamoxifen versus corn oil) on a cage by cage basis. Investigators were unaware of group assignment (‘fully blinded’) throughout data acquisition and analysis.

#### Sterile encephalitis model

Acute neurodegeneration in pyramidal neurons and subsequent neuroinflammation was induced in transgenic C57BL/6 mice double heterozygous for Neurod6^tm2.1(cre/ERT2)Kan^ (‘NexCreERT2’^25^) and Gt(ROSA)26Sor^tm1(DTA)Jpmb^ (‘Rosa26-eGFP-DTA’^26^), at the age of 7–8 months via tamoxifen. Tamoxifen was administered intraperitoneally for 3 consecutive days at a dose of 100 mg tamoxifen (CAS#10540-29-1 T5648, Sigma-Aldrich) per kilogram body weight per day. Tamoxifen was dissolved in corn oil (C8267, Sigma-Aldrich) at a dose of 10 mg/mL. Control littermates were injected with equivalent corn oil volumes.

#### Mouse monitoring

Groups of mice were inspected daily for signs of encephalitis-associated behavior (hyperactivity, circling, seizures & lethargy) in home cages. In addition, all mice were individually inspected for aberrant behavior on a weekly basis during cage changes.

#### Blood sampling

Mice were euthanized via intraperitoneal injection of 300–700 mg/kg body weight 2,2,2,-tribromoethanol (T48402, Sigma) solubilized in water. Approximately 400 µL blood was collected into syringes filled with 50 µL of 80 mM EDTA solution by cardiac puncture prior to perfusion. For blood flow cytometry, 100 µL whole blood were used immediately. Plasma was collected from the remaining blood samples after centrifugation at 1000*g* for 10 min at room temperature and stored at – 80 °C.

### Blood flow cytometry

Whole blood samples (100 µL) were transferred into flow cytometry tubes and incubated with antibody master mix (supplementary Table 1) for 15 min at room temperature. Red blood cells were lysed by 15min incubation with 2 mL ACK buffer (0.15 M NH_4_Cl, 10 mM KHCO_3_, 0.1 mM EDTA, pH 7.2–7.4) per sample. Samples were centrifuged at 300*g* for 5 min and washed with 1 mL ACK buffer. Lymphocytes were resuspended in 500 µL FACS buffer (2% BSA in PBS) and measured on a FACSymphony S6 (BD). Data acquisition was stopped after recording 30,000 APC quantification beads per sample.

Flow cytometry data was imported to FlowJo v.10.9.0 and compensated using single stained controls. An empty 540LP/35BP channel in the 445 nm Laser line was used to record autofluorescence. Manual clean up gating was performed on SSC-A versus FSC-A to separate cells and quantification beads. As eGFP is ubiquitously expressed in cells of heterozygous Rosa26-eGFP-DTA mice, lymphocytes were selected by gating of eGFP and CD45 double positive events. FSC-H versus FSC-A gating was used to exclude doublets. After individual clean-up gating, all single lymphocyte events from all samples were concatenated. Uniform hierarchical gating was performed on the concatenated sample and copied to individual samples for enumeration of cell types and subsets. First, granulocytes were identified based on high SSC-A and Ly6G. Non-granulocytes were gated for CD115 and NK1.1 to identify NK cells (NK1.1+) and monocytes (CD115+). Next, B cells were identified by gating for CD19 within the CD115 and NK1.1 negative cells, followed by gating for CD8a versus CD4 within CD19- events to identify CD8+ and CD4+ T cells respectively. Negative events were further subdivided into other myeloid cells (CD11b+, FcR+) and unclassified events (lineage negative). The quality of the hierarchical gating strategy was further evaluated qualitatively using t-SNE clustering. Major cell types were further divided into distinct subsets based on their expression of CD27, CD138, CD44, CD62L, and CD11b. The following immune cell subsets were identified: CD27+/CD11b+ M1 NK cells, CD27−/CD11b+ M2 NK cells, CD27+/CD11b− immature NK cells, and CD27−/CD11b− immature NK cells^27^, CD27-/CD138- naïve B cells; CD27+/CD138− memory B cells; CD138+ antibody secreting cells^28^; CD62L+/CD44− naive T cells, CD62L−/CD44+ effector/ effector memory T cells, and CD62L+/CD44+ central memory T cells^29,30^. For statistical analyses, the major immune cell types were normalized to the total amount of white blood cells measured per sample. To assess the differentiation state of individual cell types, immune cell subsets were normalized to their parent population (cell type of interest). Data was exported to GraphPad Prism (v 10.1.0). Data normality was assessed using Shapiro Wilk test. Groups with normally distributed data were compared using Welch’s corrected unpaired two-sided t-tests, whereas non-normal data was compared using the non-parametric Mann–Whitney tests.

### Autoantibody testing

For serological analyses, mouse blood was diluted 1:100 and tested for autoantibodies using biochip mosaics (Euroimmun, Lübeck, Germany) as previously described^31^. Briefly, these mosaics contained unfixed, nitrogen-frozen tissue cryosections (4µm from monkey cerebellum) alongside recombinant cell substrates of formalin- or acetone-fixed transfected HEK293 cells. The expression of recombinant autoantigens was confirmed using human or commercially available monospecific antibodies. The following 49 disease-associated neural antigens^19,21,23,32–53^ were evaluated: Myelin, NMDAR, AMPA-R1/R2, GABAR-B1/B2, LGI1, CASPR2, GAD65, mGluR1, mGluR5, Ma2, Recoverin, Zic-4, CARPVIII, Hu, Ri, CV2, Neurochondrin, Yo, ITPR1, Homer-3, Neurexin, ERC1, ARHGAP26, DNER, IgLON5, DPPX, AT1A3, GLRa1b, AQP4, GluRD2, MOG, GABA-a, Flotillin, KCNA1, MBP, DRD2, Contactin-2, KCNA2, Neurofascin 155, Neurofascin 186, Contactin-1, Sez6l2, AP3B2, PCA-2, AGNA, Amphiphysin, MAG, GFAP, ANNA-3. Autoantigen expression was validated by immunological methods employing monospecific control antibodies. To detect autoantibodies of the IgG, IgA and IgM isotypes, the following fluorescently labeled isotype-specific anti-mouse immunoglobulin antibodies were used: anti-mouse IgA (Cat. #62-6700, Thermo; Alexa Fluor 488 custom-labeled at Synaptic Systems), Alexa Fluor 488 labeled anti-mouse IgM (A21042, Thermo), Alexa Fluor 488 labeled anti-mouse IgG (A21202, Thermo).

### Histology

Mice were transcardially perfusion with Ringer solution and 4% formaldehyde in PBS after anesthetic euthanasia via intraperitoneal injection of 300–700 mg/kg body weight 2,2,2,-tribromoethanol (T48402, Sigma) solubilized in water. Brains were dissected and post-fixed overnight in 4% formaldehyde in PBS, dehydrated for two days in 30% sucrose solution, embedded in optimal cutting medium (Tissue-Tek, #4583, Sakura), frozen, cryosectioned, and stored in cryoprotectant solution (1× PBS with 25% ethylene glycol, 25% glycerol). Histological analyses were performed in 30-70µm coronal sections as previously described^54,55^.

*Fluorojade C staining of degenerating neurons* was performed as previously described (1, 2). Briefly, 30 µm coronal sections were mounted on microscopy slides, dried overnight at room temperature and rehydrated. Rehydrated slides were incubated with 0.06% potassium permanganate solution for 10 min, rinsed with water, and stained for 10 min in a 0.0001% solution of Fluorojade C (AG325, Sigma) and 0.1 µg/mL 4′,6-diamidino-2-phenylindole (DAPI, D9542, Sigma), dissolved in 0.1% acetic acid. Lastly, slides were rinsed with ddH_2_O, dried at 60 °C, and mounted. Images were acquired as tile scans with a pixel scaling of 1.04 µm on a Zeiss LSM880 laser scanning confocal microscope equipped with a 10× air objective (0.45NA). Presence of neurodegeneration was evaluated qualitatively by a blinded investigator. Rating criteria was the presence or absence of at least 10 distinct Fluorojade C positive cell bodies within the hippocampus.

*Iba1 and CD45* double-labeling of microglia and infiltrating lymphocytes, was performed in 30µm coronal sections using standard free-floating immunofluorescence techniques with the following antibodies: rat anti-CD45 (1:250; clone 30-F11, 103101, Biolegend), rabbit anti-IBA-1 (1:250, 019-19741, Wako), Alexa Fluor-555 anti-rabbit (1:500, A-21428, Invitrogen), Alexa Fluor 647 anti-rat (1:500, A-31571, Thermo). Images of 2–4 hippocampi per mouse were acquired as tile scans with a pixel scaling of 0.52µm on a Zeiss LSM880 laser scanning confocal microscope equipped with a 20× air objective (0.8NA). Image segmentation and quantification was performed manually with FIJI-ImageJ^56^ (version 1.54f; https://imagej.net/software/fiji/). Data from multiple hippocampi per mouse were averaged and groups were compared in Prism 10.1.0 using the Kruskal–Wallis test and the uncorrected Dunn’s test for post-hoc pairwise comparisons.

*Quantification of CNS-infiltrating T cells and B cells* were performed using triple-labeling of CD45, CD3, and CD19 in 30µm coronal brain sections. Standard free-floating immunofluorescence were used with the following antibodies: rat anti-CD45 (1:250; clone 30-F11, #103101, Biolegend), hamster anti-CD3 (1:250, clone 145-2C11, #557306, BD), rabbit anti-CD19 (1:250, clone D4V4B, #90176S, Cell Signaling), Alexa Fluor-488 anti-rat (1:500, #712-547-003, Jackson Immuno Research), Alexa Fluor-555 anti-rabbit (1:500, #A-21428, Invitrogen), Alexa Fluor-647 anti-hamster (1:500, #127-05-1685, Jackson Immuno Research). Images were acquired as tile scans with a pixel scaling of 0.69 µm on a Zeiss LSM880 laser scanning confocal microscope equipped with a 20× air objective (0.8NA). Image segmentation and quantification was performed manually with FIJI-ImageJ^56^. CD3+/CD45+ lymphocytes were quantified as T cells while CD19+/CD45+ lymphocytes were classified as B cells. Data from multiple hippocampi per mouse were averaged and groups were compared in Prism 10.1.0 using the Kruskal–Wallis test and the uncorrected Dunn’s test for post-hoc pairwise comparisons.

*CD8 and CD3* double labeling of CD8+ T cells was performed in 30µm coronal sections using standard free-floating immunofluorescence techniques with the following antibodies: hamster anti-CD3 (1:250, clone 145-2C11, #557306, BD), rat anti-CD8a (1:250, clone S18018A, #164702, Biolegend), Alexa Fluor-555 anti-hamster (1:500, A78964, Invitrogen), Alexa Fluor-647 anti-rat (1:500, A21247, Invitrogen). Images were acquired as tile scans with a pixel scaling of 0.83µm on a Zeiss LSM880 laser scanning confocal microscope equipped with a 20× air objective (0.8NA). Image segmentation and quantification was performed manually with FIJI-ImageJ^56^. CD3+/CD8+ double positive cells were counted as CD8+ T cells. Data from multiple hippocampi per mouse were averaged and groups were compared in Prism 10.1.0 using the Kruskal–Wallis test and the uncorrected Dunn’s test for post-hoc pairwise comparisons.

*Morphological analysis of microglia* was performed in 70µm coronal brain sections after staining with a standard free-floating immunofluorescence protocol using the following antibodies: rabbit anti-Iba1 (1:250, #019-19741, Wako), Alexa Fluor 555 labeled anti-rabbit (1:500, A-21428, Invitrogen). Nuclei were stained for 10 min at room temperature with 0.2 µg/mL 4′,6-diamidino-2-phenylindole (DAPI, D9542, Sigma) in PBS. Per mouse, 1–3 Z stacks spanning 20 µm (0.4 µm step size) were acquired within the *cornu ammonis* (CA) with a pixel scaling of 0.2 µm on a Zeiss LSM880 laser scanning confocal microscope equipped with a 40× oil objective (1.4 NA). All imaging settings including LASER intensities and pixel dwell times were kept constant throughout the experiment. Images were processed using the microglia morphology quantification tool (MMQT) developed by Heindl, Gesierich and colleagues^57^. Images with visible artifacts in the orthogonal views and images with poor spatial correlations in Z and XY dimensions were excluded from the analysis. Relevant parameters describing the 3D morphology of microglia were extracted and microglia were filtered using the following parameters: distance to X & Y borders > 15 µm; distance to Z borders > 5 µm; number of nuclei = 1; merged soma = 0; shortest distance between microglia soma > 15 µm; number of branches > 0. Linear mixed-effects models were used to compare morphological parameters between groups, with groups as a fixed effect and individual animals as a random effect to account for repeated measures within mice (multiple microglia per mouse). Models were fitted using the lmerTest package^58^ and p-values for fixed (group) effects were extracted in R.

### Statistical analysis

Statistical analyses were performed as described in respective method sections. Analyses were performed either in Prism 10.1.0 (GraphPad Software) or R v4.3.1^59^. Statistical tests were selected based on experimental design and data distribution. Unless stated otherwise, results are presented as mean ± standard deviations (SD). P-values < 0.05 were considered statistically significant.

## Results

### Longitudinal monitoring of mice after sterile induction of neuronal death did not reveal autoimmune encephalitis-like symptoms

**Fig. 1:**
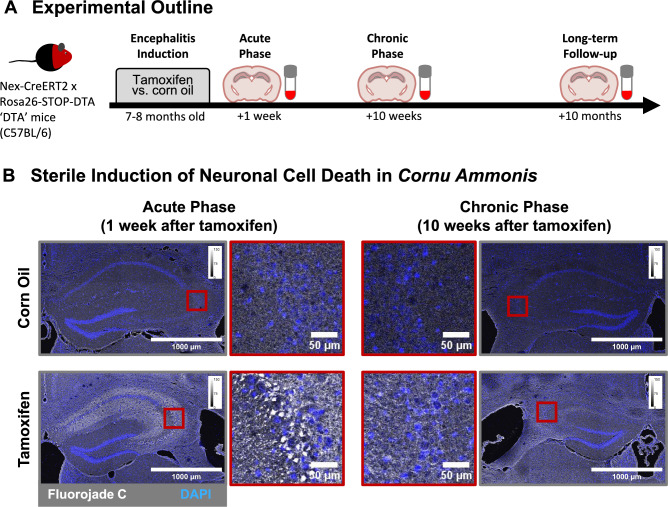
Long-term monitoring for autoimmune relapse in mice after sterile induction of acute neuronal cell death. (**A**) Experimental outline. (**B**) Fluorojade C staining of degenerating neurons, demonstrating the pronounced degeneration of neurons in the *cornu ammonis* region 1 week after tamoxifen-induced DTA expression. Neurodegeneration was absent during the recovery phase at 10 weeks post induction. Representative images of n = 4 mice per group and time point.

**Fig. 2:**
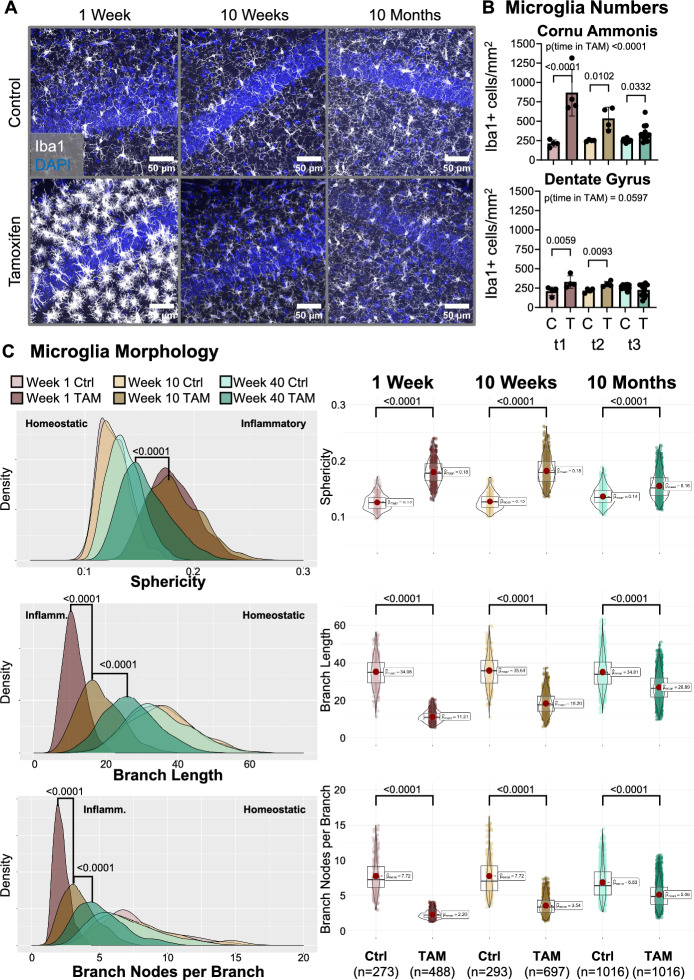
Monitoring of microglia activity at 1 week, 10 weeks, and 10 months after tamoxifen-induced neurodegeneration. (**A**) Representative maximum intensity projections of Z-stacks within the CA1 of control versus tamoxifen-induced mice at several time points. (**B**) Quantification of Iba1+ cells (microglia/macrophages) in *cornu ammonis* and dentate gyrus. Data from 4–12 mice/group and time points presented as mean ± SD. Statistical analysis was performed in Prism 10.1.0 using Kruskal–Wallis test and for pairwise comparisons the post-hoc uncorrected Dunn’s test. (**C**) Morphological analysis of microglia within the *cornu ammonis* of tamoxifen-induced (TAM) versus control (Ctrl) mice. Data from 273 to 1016 microglia of 4–12 mice/group were analyzed using linear mixed-effects models to compare morphological parameters between groups, with groups as a fixed effect and individual animals as a random effect to account for repeated measures within mice (multiple microglia per mouse).

To test if the acute destruction of neurons in the absence of any virus is sufficient to induce an autoimmune relapse in mice, we utilized a previously characterized transgenic mouse model of virus-like but sterile encephalitis^55^. Acute neurodegeneration of hippocampal and cortical pyramidal neurons and subsequent neuroinflammation was induced in adult double heterozygous NexCreERT2 x Rosa26-eGFP-DTA mice via intraperitoneal tamoxifen injections (Fig. 1A). A total of 64 mice (1:1 male/female ratio) were used for this study, of which half received tamoxifen whereas the other half received corn oil (healthy controls). To monitor disease progression histologically, 4 mice per group and timepoint were euthanized for tissue collection 1 week and 10 weeks after encephalitis induction (Fig. 1). The remaining 24 mice per group were monitored daily for autoimmune encephalitis-like symptoms, such as hyperactivity, circling, seizures, lethargy, and death^60^ for 10 months. Two corn oil control mice died spontaneously 221 days and 305 days after injections, likely due to age-related reasons (14.7 and 17.5 months). None of the tamoxifen injected mice died and autoimmune encephalitis-like symptoms were absent in all mice throughout the study period. The presence of neurodegeneration and neuroinflammation were confirmed 1 week after tamoxifen injection during the acute phase of the primary encephalitis (n = 4/ group; Fig. 1B and Fig. 2A). By ten weeks after tamoxifen induction, degenerating neurons had been cleared from the hippocampus, as indicated by the absence of Fluorojade C positive cell bodies (n = 4/group; Fig. 1B).

### Microglia profiling in the hippocampus points towards chronic neuroinflammation rather than relapse with acute autoimmune encephalitis

While there is no clear consensus on the definition of neuroinflammation yet, it is generally accepted that a key feature of neuroinflammation is the response of microglia to pathogenic stimuli, such as degenerating neurons and DAMPs or CNS infiltrating leukocytes^61^. In neurodegenerative diseases and autoimmune encephalitis, this response is typically characterized by an increase in microglia numbers/density and morphological changes^62–68^. To longitudinally monitor the inflammatory status of microglia we quantified their density and morphology 1 week, 10 weeks and 10 months after induction of acute neuronal cell death (Fig. 2A). During the acute phase, microgliosis was most prominent in the *cornu ammonis*, which contained the majority of degenerating neurons, but a significant increase of microglia numbers was also observed within the neighboring dentate gyrus (Fig. 2B). Within the *cornu ammonis*, microglia numbers remained significantly higher in tamoxifen-induced mice versus corn oil controls but significantly decreased over time (Fig. 2B). In the dentate gyrus, microglia numbers remained significantly elevated during the 10-weeks timepoint, but did not differ from control mice 10 months after tamoxifen induction. Further automated profiling of microglia 3D-morphology via MMQT^57^ revealed significant morphological changes, such as increased sphericity and decreased branching that are consistent with an inflammatory phenotype (Fig. 2C). While the microglia morphology of tamoxifen-induced mice remained significantly different from microglia in healthy control mice, we noticed a significant shift of morphological parameters towards a homeostatic morphology over time (Fig. 2C).

### Characterization of CNS-infiltrating immune cells reveals an absence of B cell and presence of CD8+ T cell infiltration

**Fig. 3:**
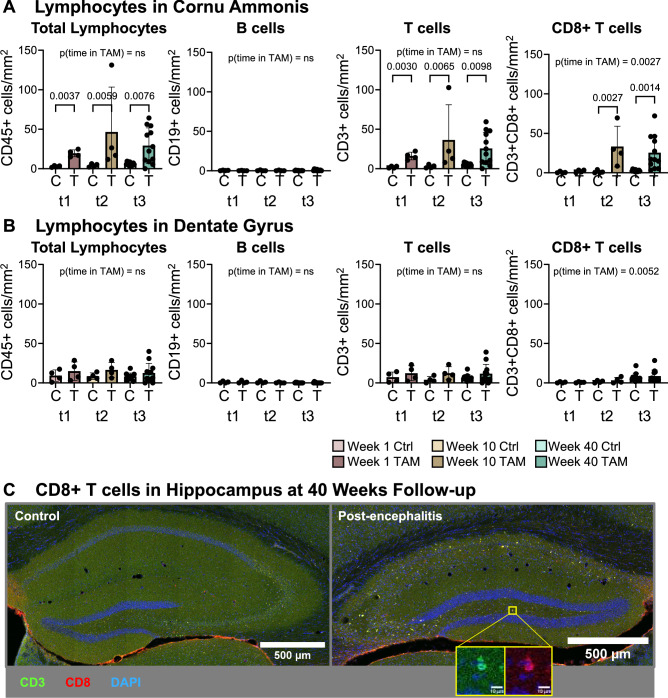
Characterization of lymphocytes within the cornu ammonis (**A**) and dentate gyrus (**B**) at different time points after tamoxifen-induced neurodegeneration (TAM, T) versus healthy control mice (Ctrl, C). t1, 1 week; t2, 10 weeks; t3, 10 months after primary encephalitis induction. Data from 4 to 12 mice/group and time point presented as mean ± SD. Statistical analysis performed in Prism 10.1.0 using Kruskal–Wallis and pairwise comparisons post-hoc uncorrected Dunn’s test. (**C**) Representative images of hippocampal CD8 T cell infiltration 40 weeks after primary encephalitis onset.

A hallmark of autoimmune encephalitis is the presence of CNS infiltrating lymphocytes, in particular B cells, T cells, and antibody secreting cells^19,63,66^. For this reason, we monitored the lymphocyte infiltration at discrete time points after primary encephalitis induction. Throughout the study period, mild to moderate significant lymphocyte infiltration was observed in the *cornu ammonis* (Fig. 3A). No significant lymphocyte infiltration was observed in the adjacent dentate gyrus (Fig. 3B). CD19 positive B cells were nearly absent in all hippocampal regions throughout the study period (Fig. 3A,B) and were not detected in noticeable amounts in other brain regions. The majority of hippocampus infiltrating CD45+ lymphocytes in tamoxifen-induced mice were CD3+ T cells (82.5% ± 14.6%; mean ± SD). While the total amount of intrahippocampal CD3+ T cells did not significantly change overtime, we noticed a significant time-dependent increase in CD8+ T cells in tamoxifen-induced mice (Fig. 3A–C). After primary encephalitis induction, the proportion of hippocampal CD8+ T cells amongst total hippocampal T cells increased from 25.0% ± 24.0% during the acute phase to 71.6% ± 29.8% during the recovery phase and 89.2% ± 14.8% after completion of the long-term follow-up. Perivascular cuffs or clusters of nuclei dense immune cell infiltrates, that are frequently present in brains of mice and humans with autoimmune encephalitis such as NMDAR encephalitis^60,63,66^, were not observed.

### Blood flow cytometry indicates absence of a peripheral autoimmune response

**Fig. 4:**
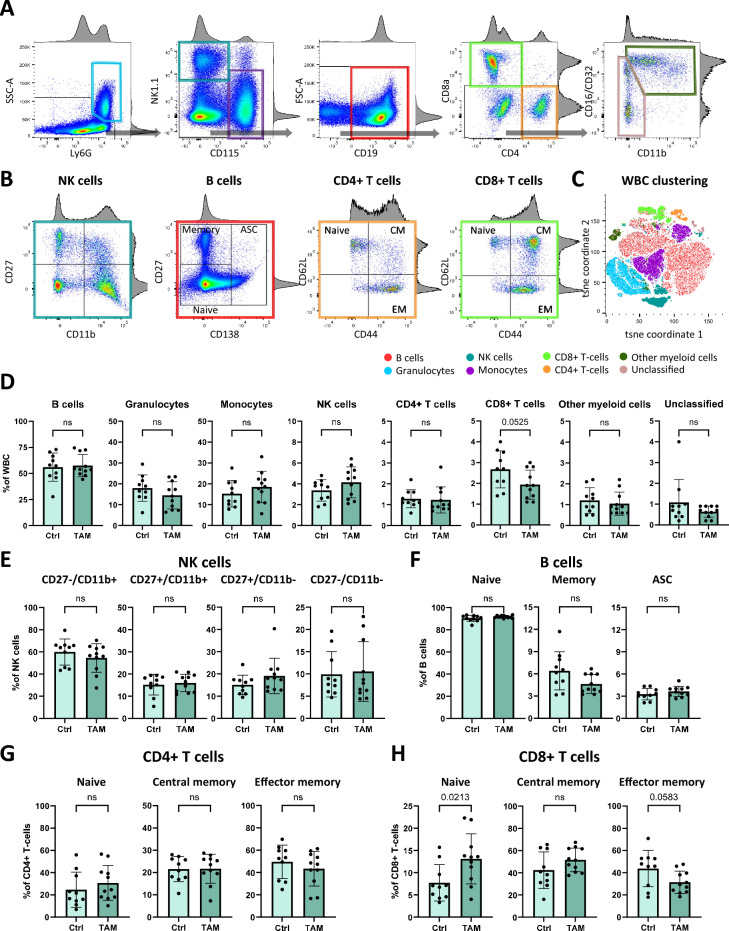
Flow cytometry of leukocytes in blood, 10 months after encephalitis induction via tamoxifen (TAM) versus healthy control (Ctrl) mice. (**A**) Gating strategy of major immune cell types. Presented data includes all live single CD45+ cells of all mice. (**B**) Immunophenotyping of major immune cell types. (**C**) Dimensionality reduction of flow cytometry parameters showing clusters of major immune cell types. (**D**) Quantification of major immune cell types. (**E**–**H**) Quantification of immune cell subsets. Data from 10–11 mice per group are presented as mean ± SD. Statistical analysis was performed in Prism 10.1.0, data normality tested using Shapiro–Wilk test. Normally distributed data was analyzed using Welch’s corrected t-tests, non-normal data using the Mann–Whitney test.

We performed high-parameter flow cytometry analyses of peripheral blood to test if mice recovering from a virus-like but sterile encephalitis show a peripheral immune response that would be indicative of an adaptive immune response and autoimmune encephalitis. Using classical cell surface markers and hierarchical gating, we quantified granulocytes, NK-cells, monocytes, B cells, CD4+ T cells and CD8+ T cells in blood samples of 11 mice tamoxifen-induced post-encephalitis mice and 10 healthy control mice after completion of the 10-months follow-up period (Fig. 4A). Within the major immune cell populations, we quantified the proportions of distinct immune cell subtypes (Fig. 4B). NK cell subsets were classified based on differential surface expression of CD11b and CD27^27^. B cells were categorized as CD27−/CD138− naïve B cells, CD27+/CD138− memory B cells, and CD138+ antibody secreting cells^28^. T cells were divided into CD62L+/CD44− naïve T cells, CD62L+/CD44+ central memory T cells, and CD62L−/CD44+ effector memory T cells^29,30^. Dimensionality reduction and t-SNE clustering was used to confirm the quality of the hierarchical gating strategy (Fig. 4C). At the time of follow-up completion, none of the major cell types were significantly altered in tamoxifen-induced post-encephalitis mice (Fig. 4D). Similarly, neither the NK cell population, B cell population, or CD4+ T cell population showed significant changes in their composition (Fig. 4E–G). However, the peripheral CD8+ T cell compartment of post-encephalitis mice showed a significant increase in naïve T cells that was accompanied by a reduction of effector memory cells (Fig. 4H).

### Testing for 49 CNS-targeting autoantibodies did not find increased autoantibody production in mice weeks to months after sterile encephalitis

CNS-directed autoantibodies are frequently observed in patients after HSE and JE^2,3,5–7,9,13,15,16^ as well as in mice infected with HSV-1^69^. To test if this autoantibody production occurs in response to acute neuronal cell death without additional co-stimulation of the immune system by virus/pathogen associated molecular patterns, we tested plasma samples of healthy mice and mice after induction of virus-like but sterile encephalitis for the presence of anti-CNS autoantibodies. Autoantibody titers against 49-disease associated antibodies were determined for IgG, IgA and IgM isotypes using commercially available cell-based assays developed for the in vitro diagnostic of autoantibodies in suspected autoimmune encephalitis patients (Euroimmun biochip mosaic IVD assays). Due to the limited amount of mouse plasma, autoantibody testing was performed with a cut-off titer of 1:100. Plasma samples of 60 mice (n = 30 per group) were tested. One healthy control had a titer of 1:320 for anti-CASPR2 autoantibodies of the IgG subtype and one post-encephalitis mouse had IgM autoantibodies against homer-3 at a titer of 1:1000 (supplementary Table 2). Overall seroprevalence for all 49 autoantibodies was low (2/60 mice; 3.33%) and no difference was observed between groups (1/30 positive mice post-encephalitis versus 1/30 positive control mice; p > 0.9999, Chi-square test).

## Discussion

To test if the acute destruction of neurons in the absence of encephalitogenic virus is sufficient to induce a secondary autoimmune encephalitis in mice, we monitored mice for up to 10 months after sterile induction of cell death in hippocampal and pyramidal neurons. Throughout the study period, we observed no symptoms of severe autoimmune encephalitis, such as hyperactivity, circling, seizures, or lethargy. Microglia analysis revealed significant microgliosis one week after encephalitis induction, which diminished over time. Scattered lymphocyte infiltration persisted in the hippocampi of encephalitis mice but did not increase significantly. Perivascular cuffs were not detected, and B cell infiltration was rare and comparable to healthy controls. High-parameter immunophenotyping of peripheral blood leukocytes did not reveal an expansion of memory B cells or antibody secreting cells, that could be indicative of an antibody-associated autoimmune response. Concomitantly, autoantibody testing against 49 neural antigens in plasma samples of post-encephalitis mice yielded mostly negative results, similar to healthy controls. Overall, these findings suggest that acute neuronal cell death and neuroinflammation alone are insufficient to trigger relapses with autoimmune encephalitis.

While we observed significant time-dependent recovery of microglial characteristics, that are typically associated with homeostatic functioning, hippocampal microglia from mice after encephalitis induction remained significantly different from healthy control microglia for up to 10 months. A potential reason for this incomplete recovery could be the accumulation of CD8+ T cells in the hippocampus of post-encephalitis mice in combination with their advanced age. In the peripheral nervous system, CD8+ memory T cells restrict axonal regeneration after spinal cord injury and mediate aging-dependent regenerative decline^70^. Within the central nervous system, CD8+ tissue-resident memory T cells are commonly observed in several neuroinflammatory and neurodegenerative conditions^71^. While CD8+ tissue resident memory Τ cells are typically induced in response to CNS infections and associated with protective properties^72^, autoreactive CD8+ tissue resident memory T cells can also drive autoinflammatory responses within the CNS^73,74^. In our model, we observed a partial time-dependent recovery of homeostatic-like characteristics in microglia as well as an absence of additional lymphocyte recruitment or encephalitis-like symptoms, pointing against a CD8+ T cell mediated autoimmune pathology.

In this study we found a lack of autoimmune encephalitis-like pathology after the induction of neuronal cell death and sterile encephalitis. A probable reason could be an insufficient stimulation of the peripheral immune system due to the absence of virus/pathogen associated molecular patterns. Several histopathological analyses of ‘sporadic’ and NMDAR encephalitis-associated ovarian teratomas highlight the relevance of peripheral immune cell activation in the encephalitogenic process. In both patient subsets, neuronal tissue, that ectopically express NMDA receptors, was frequently observed. However, ovarian teratomas from patients who progressed to autoimmune encephalitis exhibited a higher density of lymphocytic infiltrates near neuronal tissue^65,75–78^. This suggests that co-stimulatory signals play a key role in determining whether exposure to neuronal antigens is tolerated or leads to autoimmune encephalitis. The role of peripheral immune activation as risk factor for autoimmune encephalitis after virus encephalitis was further highlighted by a recent prospective study by Armangué and colleagues who found that an elevated blood IFN response was the most important predictor of post-HSE autoimmune encephalitis^7^.

In principle, the absence of an autoimmune relapse following encephalitis could also be explained by an insufficient presentation of autoantigens to the immune system. While shedding of neuronal antigens, such as NMDA and AMPA receptors, into the periphery via small vesicles has been shown to occur in human encephalitis patients^79,80^, we did not assess this shedding process in the present mouse model. Interestingly, DTA-mediated ablation of oligodendrocytes in adult mice induced the generation of myelin oligodendrocyte glycoprotein (MOG)-specific T cells in peripheral lymphoid organs and resulted in an autoimmune relapse approximately 30 weeks after oligodendrocyte ablation^24^. The apparent discrepancy between the outcome after acute pyramidal cell and oligodendrocyte cell death suggest that factors such as the antigenicity of the released proteins as well as antigen-specific clearance and shedding may determine susceptibility to autoimmune relapses.

## Limitations

A limitation of the utilized autoantibody screening is the use of commercially available cell-based assays, that were developed as in vitro diagnostics for the detection of autoantibodies in patients with (suspected) autoimmune encephalitis. While all of the assessed autoantigens are well conserved between human and mice (95 ± 5% sequence identity), we cannot rule out the presence of autoantibodies targeting mouse-specific epitopes. Similarly, low affinity autoantibodies or autoantibodies with a low plasma concentration might have been missed due to the stringent cut-off titer of 1:100. This would also explain the lower seroprevalence in comparison to our previous work, that focused on the NMDAR1-AB seroprevalence across mammals, in which samples were tested with 1:1 and 1:10 dilutions^81,82^. Future studies investigating low-affinity/low-titer autoantibodies, should also consider replacing commercially available fixed cell-based assays with in-house live cell-based assays, as these have been shown to have an enhanced sensitivity for autoantigens such as NMDA receptors^83^. Furthermore, the repertoire of known brain autoantibody targets is still expanding and their disease associations are still being uncovered^84^. Hence, despite testing against 49 well characterized brain-autoantigens, the autoantibody testing was not exhaustive and might have missed autoantibodies against more recently discovered targets such as septin-5 and septin-7^85^. So, while our results point against relapses with autoimmune encephalitis in post-encephalitis mice due to the lack of clinical symptoms, CD4 T cell and B cell infiltration, and recovery of homeostatic-like characteristics in microglia, we cannot rule out the development of a peripheral autoimmune response against brain autoantigens.

Another limitation is that we did not investigate cases with more massive and global destruction of neurons, which would likely increase the amount of neuronal autoantigens and damage associated molecular patterns that are drained to peripheral lymphoid organs, and may reach a threshold that is sufficient to induce autoimmune relapses. Furthermore, possible alterations in the immune system of individuals following herpes encephalitis might constitute influencing factors. A potential additional feature predisposing to pathogenic brain-directed autoimmunity could be damage to glial cells and the blood brain barrier^86^. In fact, we previously observed an increase in NMDAR1-autoantibodies in mice in response to a small standardized cryolesion of the right parietal cortex^82^ with local damage of all cells near the lesion site and blood–brain barrier dysfunction^87^.

## Conclusions

While clinical observations suggest a solid link between autoimmune encephalitis and virus encephalitides, such as HSE and JE, the pathomechanisms behind the secondary autoimmune response have remained obscure. The present study on a sterile encephalitis model reveals that acute destruction of neurons alone is not sufficient to induce an autoimmune response against neuronal surface antigens, as previously associated with autoimmune encephalitides. This observation indicates that co-factors are required for the initiation of anti-neuronal autoimmune responses. Identifying these co-factors may ultimately enable targeted therapies.

## Supplementary Information

Supplementary Tables.

## Data Availability

The code used to analyze MMQT output files is available at https://github.com/vgastaldi/MMQT-longDTA. Further information and requests for data and resources should be directed to the lead contact, Prof. Dr. Dr. Hannelore Ehrenreich (hannelore.ehrenreich@zi-mannheim.de).
