# Supplementary material for: Acute neuronal cell death and neuroinflammation per se do not trigger secondary autoimmune encephalitis in mice
Source: Sci Rep. 2025 Jun 27;15:20337. doi: 10.1038/s41598-025-08035-w (PMC12205092; doi:10.1038/s41598-025-08035-w)
Supplement: Supplementary file 1 — Supplementary Tables. [file 41598_2025_8035_MOESM1_ESM.docx]

Supplementary Table 1: Blood flow cytometry antibody master mix

| **RRID** | **Marker** | **Fluorochrome** | **Clone** | **Cat#** | **Company** | **Vol (µL)** |
| --- | --- | --- | --- | --- | --- | --- |
| AB_2651134 | CD45 | BUV395 | 30-F11 | 565967 | BD | 0.25 |
| AB_2874241 | CD8a | BUV661 | 53-6.7 | 750023 | BD | 0.25 |
| AB_2871390 | CD138 | BB700 | 281-2 | 742124 | BD | 0.5 |
| - | CD44 | BUV737 | IM7 | 612799 | BD | 0.5 |
| - | CD4 | BUV563 | RM4-5 | 569182 | BD | 0.25 |
| AB_2743990 | CD62L | BV480 | MEL-14 | 746726 | BD | 0.25 |
| AB_2740505 | CD16/32 | BV786 | 2.4G2 | 740851 | BD | 0.5 |
| AB_396958 | CD19 | AF700 | 1D3 | 557958 | BD | 0.25 |
| AB_2565547 | CD27 | BV421 | LG.3A10 | 124223 | Biolegend | 0.5 |
| AB_2562679 | CD115 | BV711 | AFS98 | 135515 | Biolegend | 1 |
| AB_1133989 | CD103 | PE | 2E7 | 121406 | Biolegend | 1 |
| AB_312793 | CD11b | PE-Cy5 | M1/70 | 101210 | Biolegend | 0.5 |
| AB_389364 | NK1.1 | PE-Cy7 | PK136 | 108714 | Biolegend | 0.5 |
| - | Ly6G | AF750 | 1A8 | FAB10371S-100UG | Rnd systems | 0.5 |
| CaliBRITE beads | | APC | LOT 20131 | 340487 | BD | 5 |
| Brilliant stain buffer | | na | na | 563794 | BD | 10 |
| DAPI | | DAPI | na | D9542 | Sigma | 0.02ug |

Supplementary Table 2: Autoantibody test results

| **Animal ID** | **Group** | **Timepoint** | **Autoantibody results** |
| --- | --- | --- | --- |
| 1169 | Control | 1-week | negative |
| 1170 | Control | 1-week | negative |
| 1171 | Control | 1-week | negative |
| 1172 | Control | 1-week | negative |
| 1161 | Control | 10-weeks | negative |
| 1162 | Control | 10-weeks | negative |
| 1163 | Control | 10-weeks | negative |
| 1164 | Control | 10-weeks | negative |
| 1109 | Control | 10-months | negative |
| 1110 | Control | 10-months | negative |
| 1115 | Control | 10-months | negative |
| 1116 | Control | 10-months | negative |
| 1117 | Control | 10-months | negative |
| 1118 | Control | 10-months | negative |
| 1125 | Control | 10-months | negative |
| 1143 | Control | 10-months | negative |
| 1166 | Control | 10-months | negative |
| 1173 | Control | 10-months | negative |
| 1174 | Control | 10-months | negative |
| 1176 | Control | 10-months | negative |
| 1178 | Control | 10-months | negative |
| 1179 | Control | 10-months | negative |
| 1180 | Control | 10-months | negative |
| 1181 | Control | 10-months | negative |
| 1188 | Control | 10-months | negative |
| 1196 | Control | 10-months | negative |
| 1233 | Control | 10-months | negative |
| 1234 | Control | 10-months | 1:320 IgG anti-CASPR2 |
| 1263 | Control | 10-months | negative |
| 1264 | Control | 10-months | negative |
| 1183 | Sterile Encephalitis | 1-week | negative |
| 1184 | Sterile Encephalitis | 1-week | negative |
| 1185 | Sterile Encephalitis | 1-week | negative |
| 1186 | Sterile Encephalitis | 1-week | negative |
| 1182 | Sterile Encephalitis | 10-weeks | negative |
| 1189 | Sterile Encephalitis | 10-weeks | negative |
| 1190 | Sterile Encephalitis | 10-weeks | negative |
| 1191 | Sterile Encephalitis | 10-weeks | negative |
| 1108 | Sterile Encephalitis | 10-months | negative |
| 1126 | Sterile Encephalitis | 10-months | 1:1000 IgM anti-Homer-3 |
| 1136 | Sterile Encephalitis | 10-months | negative |
| 1141 | Sterile Encephalitis | 10-months | negative |
| 1142 | Sterile Encephalitis | 10-months | negative |
| 1144 | Sterile Encephalitis | 10-months | negative |
| 1145 | Sterile Encephalitis | 10-months | negative |
| 1150 | Sterile Encephalitis | 10-months | negative |
| 1151 | Sterile Encephalitis | 10-months | negative |
| 1241 | Sterile Encephalitis | 10-months | negative |
| 1242 | Sterile Encephalitis | 10-months | negative |
| 1265 | Sterile Encephalitis | 10-months | negative |
| 1271 | Sterile Encephalitis | 10-months | negative |
| 1272 | Sterile Encephalitis | 10-months | negative |
| 1273 | Sterile Encephalitis | 10-months | negative |
| 1281 | Sterile Encephalitis | 10-months | negative |
| 1282 | Sterile Encephalitis | 10-months | negative |
| 1283 | Sterile Encephalitis | 10-months | negative |
| 1284 | Sterile Encephalitis | 10-months | negative |
| 1285 | Sterile Encephalitis | 10-months | negative |
| 1286 | Sterile Encephalitis | 10-months | negative |
| 1287 | Sterile Encephalitis | 10-months | negative |
